# Fabrication of Medium Mn Advanced High-Strength Steel with Excellent Mechanical Properties by Friction Stir Processing

**DOI:** 10.3390/mi15081052

**Published:** 2024-08-21

**Authors:** Yonggang Yang, Wangnan Zuo, Yu Liu, Yunzong Ge, Zhiqiang Yang, Jiansheng Han, Zhenli Mi

**Affiliations:** 1 National Engineering Research Center for Advanced Rolling and Intelligent Manufacturing, University of Science and Technology Beijing, Beijing 100083, China; yangyg@ustb.edu.cn (Y.Y.);; 2 Central Iron & Steel Research Institute, Beijing 100081, China; 3 Technology Center, Ningbo Iron & Steel Co., Ltd., Ningbo 315800, China; 4 Design and Research Institute Co., Ltd. of University of Science and Technology Beijing, Beijing 100083, China

**Keywords:** manufacturing technology, medium Mn steel, microstructure, mechanical properties

## Abstract

Friction stir processing (FSP) manufacturing technology was used to fabricate medium Mn advanced high-strength steel in this study. The mechanical properties and microstructure of the steel fabricated using FSP were investigated. The steel obtained a total elongation of 35.1% and a tensile strength of 1034.6 MPa, which is about 59% higher than that of the steel without FSP. After FSP, a gradient structure occurs along the thickness direction. Specifically, across the thickness direction from the base material zone to the transition zone and finally to the stirring zone, both the grain size and austenite fraction decrease while the dislocation density increases, which results from the simultaneous effect of severe plastic deformation and recrystallization during FSP. Due to the gradient structure, an obvious difference in the strain across the thickness direction of the steel occurs during the deformation process, resulting in significant hetero-deformation-induced (HDI) strengthening. The deformation mechanism analysis reveals that HDI strengthening and dislocation strengthening are the main factors in the improvement in the strength–ductility balance. The obtained knowledge sheds light on the process of fabricating medium Mn steels with excellent properties using FSP manufacturing technology.

## 1. Introduction

Medium manganese steels (MMnS) containing 3 wt.%–12 wt.% Mn have become a candidate for use in automobile bodies and micro-/nano-devices due to their excellent strength–ductility synergy [1,2]. However, like other metallic materials, medium Mn steels lose their advantage of excellent ductility, while achieving higher strength, if manufactured using a conventional method, for example, intercritical annealing treatment, resulting in a well-known strength–ductility balance dilemma [3,4].

In recent years, efforts have been made to tailor gradient structures using novel manufacturing technologies to solve the strength–ductility trade-off problems in many metallic materials, including steels [5,6,7,8]. Wang et al. [9] used pre-torsion and annealing treatments to achieve the required grain-size gradients in low-carbon steel, and the steel obtained the optimal synergy of high yield strength, uniform ductility, and toughness. Moreover, as reported by Wei et al. [10], a gradient nanotwin structure in twinning-induced plasticity (TWIP) steel can double the steel’s yield strength without reducing its ductility. Different from grain-size gradients and nanotwins, the gradients in austenite fractions significantly improve the yield strength of stainless steel, and the yield strength increases from 502 MPa to 1010 MPa [11]. In addition to the steels above, the gradient structure, formed during the manufacturing process, has also been altered to elevate the mechanical properties of medium Mn steels. Zhang et al. [12] adopted pre-twisting and intercritical annealing manufacturing technology to tailor the gradients in austenite fractions. Compared to steel with a homogenous microstructure, the yield strength and total elongation of the gradient-structured sample increased by 49% and 68%, respectively. To date, there have been a few studies on the gradient structures in medium Mn steels, and in these studies, cold-deformation manufacturing technology was employed to fabricate the microstructural gradients [13]. However, studies on gradient structure using hot deformation are scarce.

Friction stir processing (FSP) is a hot-deformation manufacturing technology developed from friction stir welding. During FSP treatment, severe plastic deformation and high temperatures simultaneously occur in the stirring zone, and thus, the grain size and phase fraction can be tuned [14]. Moreover, similar to the stirring zone after the FSP process, the stirring zone in medium Mn steel after friction stir welding obtains a smaller grain size compared to the initial microstructure, and the austenite volume fraction increases from 42% to 49% [15]. These studies imply that the potential gradient in the grain size and phase fraction can form across the thickness direction of medium Mn steel when the thickness of the stirring zone is less than the total thickness of the steel. However, the formation mechanism for the potential gradient structure when using FSP manufacturing technology, and its effect on the mechanical properties of steel, have not been investigated.

Therefore, in the present study, a gradient structure was tailored in medium manganese steel using FSP manufacturing technology, and the microstructural evolutions during the formation of the gradient structure and the changes in the mechanical performance of the material were analyzed.

## 2. Materials and Methods

### 2.1. Materials

A 50 kg ingot of Fe-0.12C-4.37Mn (P and S below 0.05%, wt.%) steel was melted using a vacuum induction furnace. The ingots were homogenized at 1200 °C for 10 h and then hot-rolled to the plates (width—70 mm; thickness—6 mm) with a starting rolling temperature of 1150 °C and a final rolling temperature of 900 °C. After hot rolling, the steel was water-cooled to room temperature. The austenite starting temperature (A_1_) and austenite finishing temperature (A_3_) of MMnS calculated by Thermo-calc 2021b were 540.3 °C and 740.1 °C, respectively. According to the calculated results, the hot-rolled plate was heated to 650 °C for a duration of 6 h for annealing to obtain an optimal ferrite + austenite microstructure [16] and then air-cooled to room temperature to serve as the base material.

**Figure 1:**
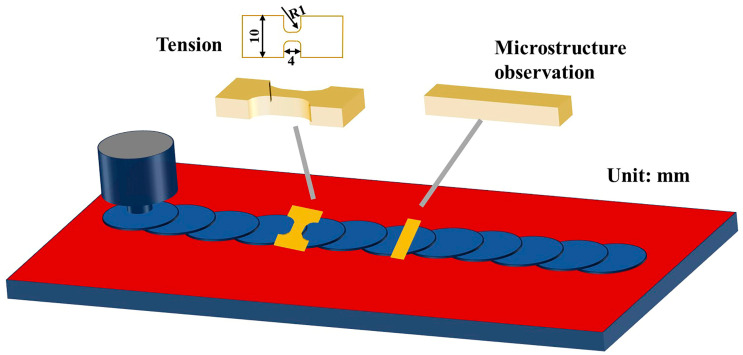
Schematic diagram of FSP and sampling position.

A friction stir welding machine (FSW, China FSW center, Beijing, China) was used to perform FSP on the annealed plates, which had dimensions of 100 mm (length) × 70 mm (width) × 6 mm (thickness), as shown in Figure 1. A W-25Re (wt.%) tapered thread stirring pin with a shoulder diameter of 15 mm, a root diameter of 8 mm, and a height of 2.7 mm was used. FSP was conducted at a constant rotation rate of 150 rpm and a welding speed of 150 mm/min. Since the height of the stirring pin was lower than the total thickness of the plate, the whole thickness of the plate was not stir-processed. After FSP, the surface of the plates was machined to reduce the thickness by 1 mm to ensure surface quality. The final thickness of the plates was 5 mm. For convenience, the specimens after friction stir processing are named FSPed samples.

Finite element analysis (FEA) was used to simulate FSP processing with the FEA model constructed using the arbitrary Lagrange–Euler method. The workpiece dimensions and the stirring pin size were consistent with the experimental setup. Hexahedral meshes were used to discretize the workpiece, with mesh refinement in the stirring zone, to prevent computational issues caused by mesh distortion during deformation. The workpiece featured 74,851 nodes and 63,126 elements. The workpiece’s movement was constrained in all directions, and contact was established between the stirring head and the workpiece. The heat-transfer coefficient between the workpiece and the tool pin, the heat-transfer coefficient between the workpiece and the environment, and the friction coefficient were set to 1000 W/(m^2^·°C), 30 W/(m^2^·°C), and 0.3, respectively [17].

### 2.2. Microstructure Characterization

The macroscopic features of the FSPed specimens were investigated using a confocal laser scanning microscope (CLSM, Olympus, Tokyo, Japan). The phase fraction and morphology of the material were characterized with a field-emission scanning electron microscope (FE-SEM, Zeiss, Oberkochen, Germany) and electron backscatter diffraction (EBSD, Oxford instruments, Oxford, UK). A transmission electron microscope (TEM, JEOL, Tokyo, Japan) equipped with energy-dispersive spectroscopy (EDS) was utilized to observe the microstructure of FSPed specimens. CLSM and SEM samples were mechanically polished and then etched with 4% nitric acid. The EBSD samples were ground and then electropolished with a mixed solution of 10% perchloric acid (HClO_4_) and 90% glacial acetic acid (CH_3_COOH) at a voltage of 20 V for 15 s. The acceleration voltage, step size, work distance, and inclination angle for EBSD measurements were 20 kV, 0.15 µm, 15 mm, and 70°, respectively. EBSD data were cleaned using AztecCrystal 2.1 software to remove zero resolution points (zero resolution < 10%) before further analysis. Grain size was calculated according to the E2627 standard [18]. TEM thin foils were prepared by double-jet electropolishing using a solution of 5% perchloric acid and 95% ethanol.

The phase fraction and dislocation density of the test steel were determined using X-ray diffraction (XRD, Bruker Corporation, Berlin, Germany). Diffraction patterns were obtained using Cu-Kα radiation (wavelength: 1.5418 Å). The scanning range for austenite volume fraction and dislocation density measurements were 40~100° and 35~145°, respectively, both at a rate of 1°·min^−1^. The austenite volume fraction was calculated from the integrated intensities of three FCC diffraction peaks (*γ*) and two BCC diffraction peaks (*α*) [19]. In addition, the average C content in austenite was estimated using Equation (1) [20]:

(1) $$\alpha_{\gamma }=3.566+0.0453\chi_{c}+0.00095\chi_{Mn}+0.0056\chi_{Al}$$

where $\alpha_{\gamma }$ represents the austenite lattice parameter. $\chi_{c}$, $\chi_{Mn}$, and $\chi_{Al}$ represent the concentrations of C, Mn, and Al in austenite (wt.%), respectively.

Dislocation densities of *α* and *γ* were determined using the modified Williamson-Hall (W-H) method [21,22].

(2) $$∆K\tilde{=}\frac{0.9}{D}+{\left(\frac{\pi A^{2}b^{2}}{2}\right)}^{1/2}\rho^{1/2}K{\bar{C}}_{hkl}^{1/2}$$

where $\Delta K=\frac{2cos\theta }{\lambda }\Delta \theta$ and $K=\frac{2sin\theta }{\lambda }$ represent the FWHM and exact Bragg position in reciprocal space, respectively; θ is the associated Bragg angle; and λ denotes the wavelength of the X-ray. *D* and *A* denote the average grain size and a constant related to the effective outer cutoff radius of the dislocation, respectively; *b* represents Hamburger vector. ρ represents dislocation density; ${\bar{C}}_{hkl}$ is the average contrast factor of dislocations [23].

### 2.3. Mechanical Properties Tests

A micro-Vickers hardness tester (HMAS-D1000 SMZ, YanRun, Shanghai, China) was employed to measure the hardness distribution in the cross-section of sample before and after deformation, using a 100 gf load for 10 s, with 0.2 mm spacing between indentations.

Tensile tests were performed on dog-bone-shaped specimens (length of the tensile zone—2 mm; width—2 mm; thickness—5 mm; total length—34 mm) perpendicular to the processing direction, as shown in Figure 1. The thickness of the tensile specimens equaled the total thickness of FSPed specimens. For comparison, the tensile specimens of the same dimension were prepared from the base material. The tensile tests were conducted on a universal testing machine (CMT5105, Sans, Shenzhen, China) at room temperature with an initial strain rate of 0.0025 s^−1^. Additionally, interrupted tensile tests were conducted by controlling the displacements. After deformation to a specific displacement, the equivalent true strain was calculated using the method described in [24].

(3) $$\varepsilon_{i}^{eq}=\frac{2}{\sqrt{3}}ln\left[-ln\frac{t_{i}}{t_{0}}\right]$$

where $\varepsilon_{i}^{eq}$ is the equivalent true strain, $t_{i}$ represents the thickness of a certain position *i* on the gauge section of the sample after tensile deformation, and $t_{0}$ denotes the initial thickness of the specimen. For accuracy, three repeated tests were performed for both hardness and tensile tests.

## 3. Results

### 3.1. Microstructure of the FSPed Steel

**Figure 2:**
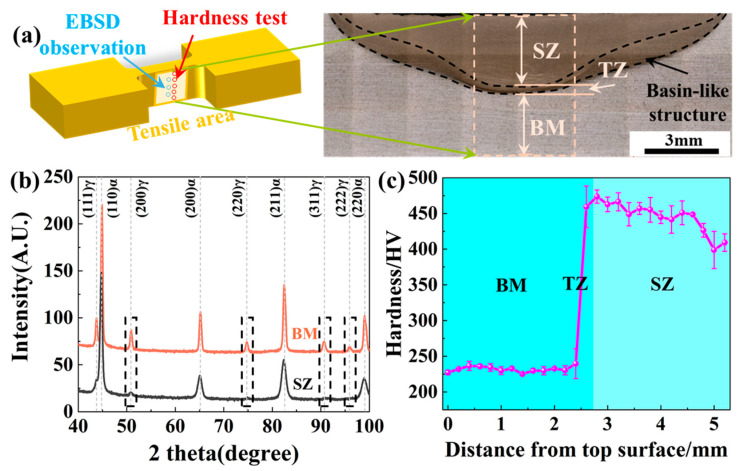
Macrostructure morphology (**a**), XRD patterns (**b**), and the hardness profile (**c**) of the FSPed sample.

Figure 2 shows the macrostructure morphology, XRD patterns, and hardness distribution of the FSPed sample. The FSPed sample exhibits a distinct stirring zone (SZ), a narrow transition zone (TZ), and a base material zone (BM), as shown in Figure 2a. A noticeable basin-like structure is present, with no defects, for example, pores, observed. The thicknesses of SZ, TZ, and BM are 2.3 mm, 0.4 mm, and 2.3 mm, respectively. In the XRD patterns in Figure 2b, both fcc and bcc peaks are observed in the SZ and BM. However, the peak intensities of the fcc phase in the SZ are lower than those in the BM. The determined austenite volume percentages in the BM and SZ are 8.7% and 2.2%, respectively, indicating a reduction in the austenite fraction after FSP. The Vickers hardness profile across the thickness direction (Figure 2c) shows that BM is relatively homogeneous, with minimal variation in hardness, and the average Vickers hardness is approximately 231.4 HV. Different from BM, the hardness in the TZ gradually increases, reaching its highest value at the TZ/SZ boundary. After reaching the highest value, the hardness decreases toward the upper surface of the FSPed steel, with an average hardness of 445.9 HV in the SZ. The hardness profile across the whole thickness direction indicates the formation of a clear gradient structure after FSP.

**Figure 3:**
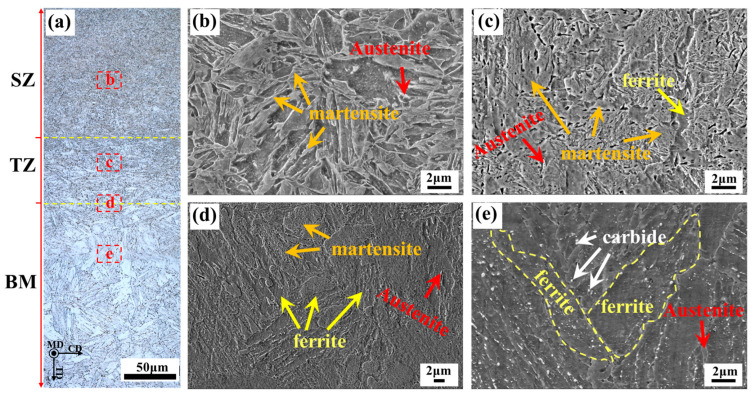
Macrostructure and microstructure morphology of FSPed sample. (**a**) Macrostructure of the steel; (**b**) Microstructure of SZ; (**c**) Microstructure of TZ; (**d**) Microstructure of TZ/BM boundary; (**e**) Microstructure of BM.

Microstructures of different regions of the FSPed sample are provided in Figure 3. It can be seen in Figure 3a that the grain size gradually decreases from the BM to the TZ and further into the SZ. In the enlarged figure (Figure 3b,c), the SZ predominantly consists of lath-like martensite with a small amount of austenite, while the TZ is composed of martensite, ferrite, and austenite. At the TZ/BM boundary (Figure 3d), martensite, ferrite, and austenite are also present, with an increased volume fraction of large-sized ferrite compared to the TZ. The BM microstructure, shown in Figure 3e, contains ferrite, fine austenite, and a small amount of carbide. The formation of carbides is related to austenite decomposition due to the slow cooling rate [25]. Compared to the BM, the occurrence of martensite in the TZ and SZ indicates that obvious martensitic transformation occurs during the cooling process after FSP treatment.

**Figure 4:**
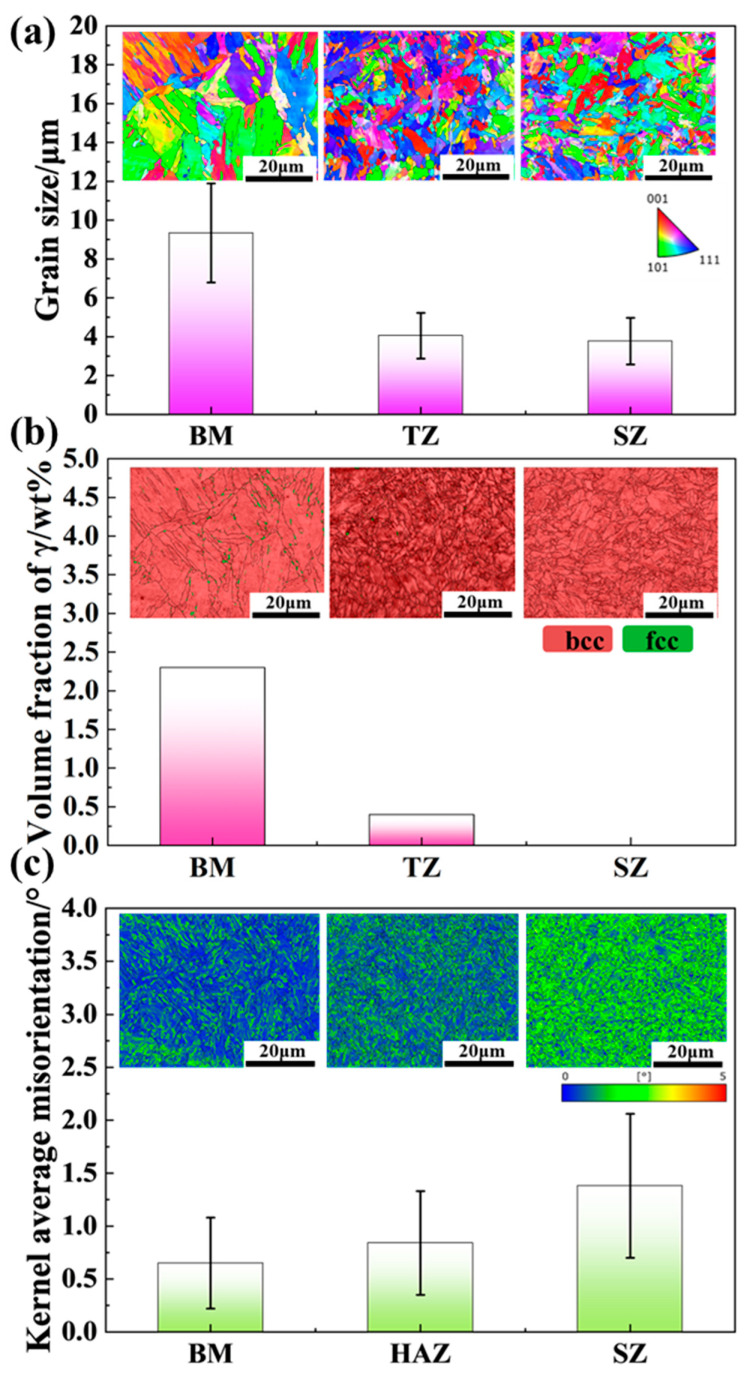
EBSD maps of BM, TZ, and SZ in the cross-section of the FSPed sample. (**a**) Average grain size and IPF maps; (**b**) Austenite volume fraction and BC+ phase maps; (**c**) Kernel average misorientation (KAM) value and KAM maps.

Figure 4 shows the inverse pole figure (IPF) maps, band contrast (BC)+ phase maps, and kernel average misorientation (KAM) maps of the BM, TZ, and SZ regions of the FSPed sample. The IPF maps in Figure 4a reveal that the grain morphology transitions from lath-like in BM to block-like in TZ and SZ. According to the effective grain size distribution maps (Figure S1 in the Supplementary Materials), the calculated effective grain sizes are 9.0 μm for BM, 4.1 μm for TZ, and 3.8 μm for SZ, indicating a grain size gradient along the thickness direction. The Band contrast (BC)+ phase maps in Figure 4b show that both BM and TZ consist of the bcc phase with a small amount of fcc, while SZ primarily consists of the bcc phase. The austenite fractions, as determined by EBSD, decrease from BM (2.3%) to TZ (0.4%) and to SZ (0%), indicating a slight gradient in austenite fraction across the thickness direction. Although the EBSD results for austenite fraction show a consistent trend with the XRD results (Figure 2b), the values differ. As indicated by the arrows in the BC and phase maps (Figure S2 in the Supplementary Materials), relatively low band contrast values and small austenite grains are observed in BM and SZ. The discrepancy in austenite fraction between the EBSD and XRD results likely arises from the difficulty in indexing thin austenite from surrounding phases using EBSD [23]. Figure 4c shows that the KAM value increases significantly from BM to TZ and further to SZ, with KAM values of 0.65°, 0.84°, and 1.38°, respectively, indicating a clear KAM gradient along the thickness direction.

**Figure 5:**
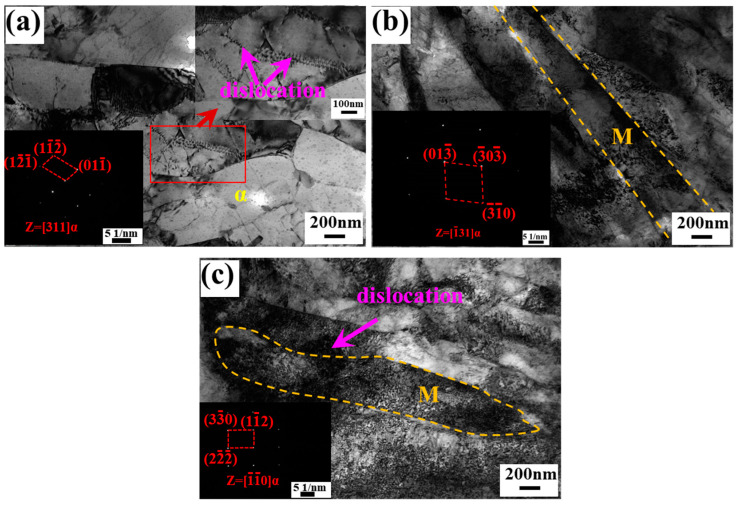
TEM images of BM, TZ, and SZ of FSPed sample. (**a**) BM; (**b**) TZ; (**c**) SZ. α: ferrite; M: martensite.

TEM images of the microstructure in the BM, TZ, and SZ regions of the FSPed sample are shown in Figure 5. Coarse ferrite is observed in BM (Figure 5a), with distinct dislocation lines present in the ferrite. In TZ (Figure 5b), lath martensite is visible, accompanied by dislocation tangles within the martensite. Similar features are observed in SZ (Figure 5c), though with a higher dislocation density. The dislocation density of bcc phases in BM and SZ, calculated using the W-H method, are 3.4 × 10^14^ m^−2^ and 11 × 10^14^ m^−2^, respectively. However, the dislocation density of TZ could not be determined due to difficulties in obtaining its XRD profile. The geometrically necessary dislocation (GND) density in the three zones was determined using KAM values, calculated by the following formula [26,27]:

(4) $$\rho_{GND}=\frac{2\omega }{\mu b_{perfect}}$$

where *ω* denotes the average misorientation angle between a measurement point and the nearest neighboring points. μ represents the step size with a value of 150 nm, and $b_{perfect}$ represents the Burgers vector of dislocation in the bcc phase, which equals 0.248 nm. The dislocation density of the bcc phase in BM, TZ, and SZ were determined to be 5.1 × 10^14^ m^−2^, 7.9 × 10^14^ m^−2^, and 9.05 × 10^14^ m^−2^, respectively. Although there are slight differences between the dislocation density values obtained from XRD and EBSD due to the different methods used, both results suggest the same trend: BM has a lower dislocation density than SZ, indicating a gradient in dislocation density across the thickness direction.

### 3.2. Microstructure of the FSPed Steel after Deformation

**Figure 6:**
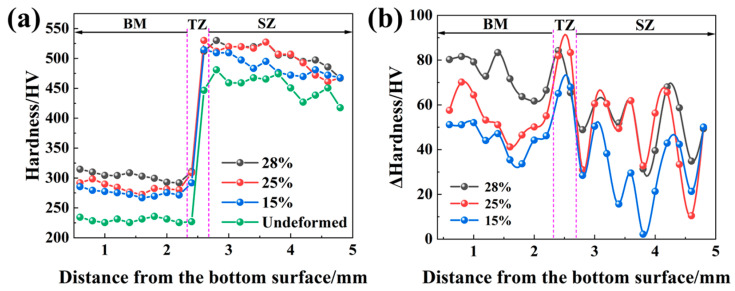
Hardness profile across the thickness direction and hardness increment under different equivalent true strains (0%, 15%, 25%, and 28%). (**a**) Hardness distribution; (**b**) Hardness increments.

Figure 6 shows the hardness distribution across the thickness direction of the FSPed specimen at different strain levels. The hardness profile of the specimen before deformation is also provided in Figure 6a. All profiles exhibit a similar trend: relatively stable hardness values in the BM, rapidly increasing hardness in the TZ, and slightly decreasing hardness in the SZ. Moreover, as the deformation degree of the specimen increases, almost all positions across the thickness direction exhibit an increase in hardness, indicating the three zones deform simultaneously in the specimen. The hardness increment, calculated as the difference between the hardness at a given strain and the hardness before deformation, is shown in Figure 6b. It can be seen that all zones exhibit hardness increments, but the increments vary across the thickness direction. This indicates that the degree of deformation varies across the thickness direction during the deformation process. At all strain levels, the hardness increments near the TZ/BM and TZ/SZ boundary are slightly higher than those in the BM and SZ. This suggests hetero-deformation near the boundaries in the TZ and hetero-deformation-induced stress in the TZ.

**Figure 7:**
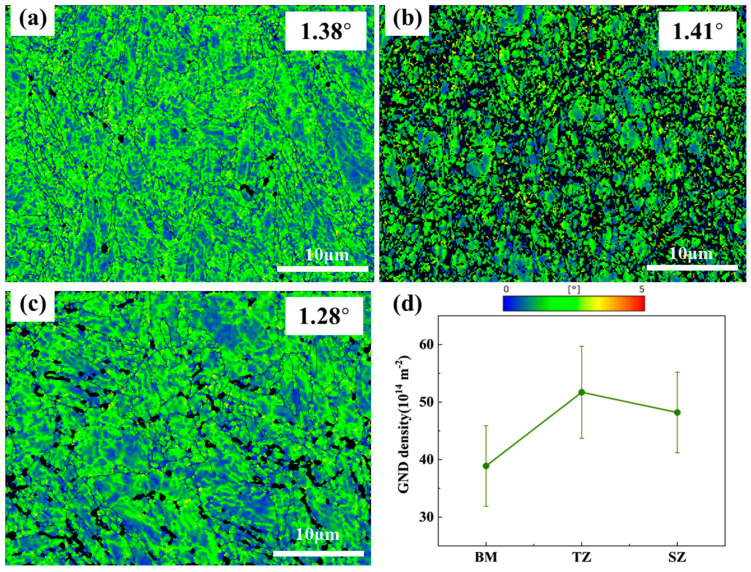
KAM maps of BM, TZ, and SZ of the FSPed specimen after an equivalent true strain of 0.28. (**a**) BM; (**b**) TZ; (**c**) SZ; (**d**) determined GND values.

KAM maps of the BM, TZ, and SZ regions of the FSPed specimen after an equivalent true strain of 0.28 are displayed in Figure 7. The BM shows a KAM of 1.38°, indicating slight deformation (Figure 7a), while the TZ exhibits a dark green KAM map with an average KAM value of 1.41° (Figure 7b), suggesting much more severe deformation. Compared to the BM and TZ, the SZ exhibits lower deformation with an average KAM value of 1.28° (Figure 7c). The calculated dislocation densities of the BM, TZ, and SZ are 39 × 10^14^ m^−2^, 52 × 10^14^ m^−2^, and 48 × 10^14^ m^−2^, respectively. These calculated dislocation density values correlate well with those reported in other studies involving friction stir welding conditions [15,28,29]. In this study, as tensile deformation increased, the GND dislocation density also significantly increased. Additionally, this result is consistent with the KAM value sequence, implying that TZ has the highest degree of deformation.

### 3.3. Tensile Property

**Figure 8:**
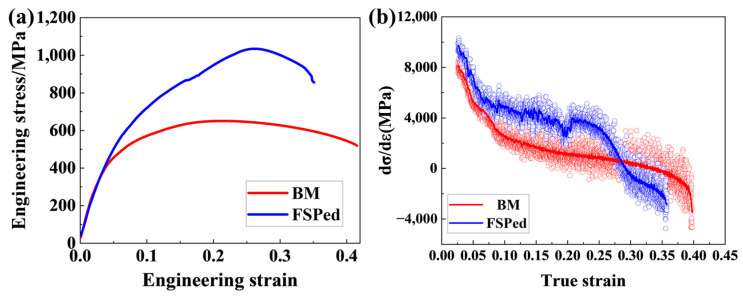
Engineering stress–strain curves and work hardening rate–true strain curves of the base material and FSPed specimens. (**a**) Engineering stress–engineering strain curves; (**b**) Work hardening rate–true strain curves.

Figure 8 shows the engineering stress–strain curves and work hardening rate–true strain curves of the base material and FSPed specimens. The engineering stress–strain curves show continuous yielding behavior during the tensile process of the specimens with and without FSP (Figure 8a). The yield strength (YS), ultimate tensile strength (UTS), and total elongation (TE) of the base material are 325.1 ± 17.1 MPa, 650.7 ± 28.2 MPa, and 38.3 ± 2.4%, respectively. After FSP, the YS and UTS of the FSPed sample increase dramatically to 380.8 ± 21.9 MPa and 1034.6 ± 19.4 MPa, respectively. Compared with the base material, the ultimate tensile strength of the FSPed specimen significantly improves while maintaining a high level of ductility (35.1%). As a result, the product of tensile strength and elongation of FSPed samples increased significantly from 24.9 GPa∙% to 36.3 GPa∙%. In Figure 8b, it can be found that the base material and FSPed specimens have similar work hardening rate curves. Both work hardening rate curves gradually decrease from a true strain of 0.04. In comparison with the base material case, the work hardening rate of the FSPed sample is much higher in the strain range of 0.04~0.28. With the increasing strain (>0.28), the work hardening rate of the FSPed specimen drops sharply, while the base material has a relatively stable decrease.

## 4. Discussion

### 4.1. The Formation Mechanism of the Gradient Structure during the Manufacturing Process

**Figure 9:**
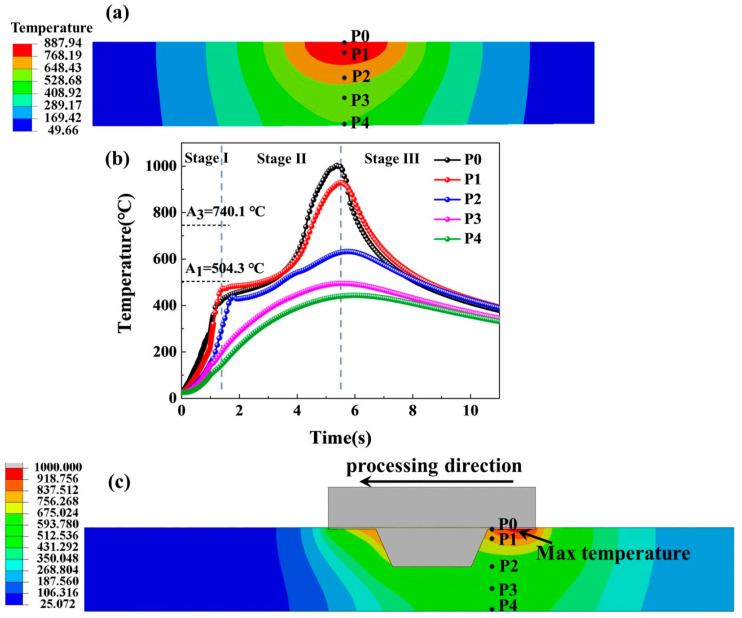
Simulated results of temperature field during FSP process. (**a**) Longitudinal section map; (**b**) Temperature–time curves; (**c**) Cross-section map.

A gradient structure, including grain size gradient, austenite fraction gradient, and dislocation density gradient along the thickness direction, has formed in the investigated MMnS after FSP. Different from the cold-deformation manufacturing technologies used to fabricate the gradient structures in other materials, such as surface mechanical attrition treatment (SMAT) [30], ultrasonic severe surface rolling (USSR) [11], and pre-torsion [31], the gradient structure in this study is formed during the hot-deformation process. To better understand the FSP process, a finite element simulation was performed to illustrate the temperature field. As shown in the longitudinal section map of the FSPed specimen (Figure 9a), an obvious temperature gradient appears across the thickness direction during FSP, and thus SZ, TZ, and BM zones form. Five points were selected to show the total temperature–time curves during the FSP (Figure 9b), which represent the temperature change of SZ (P0, P1), TZ (P2), and BM (P3, P4), respectively. All the points have three-stage temperature changes during FSP. Firstly, the temperatures of the points increase rapidly to a certain value, then slowly increase when the tool pin does not reach the points. As the tool pin continues to move, the temperatures continue to rise, reaching a peak when the tool pin arrives at the points. Compared to the P0, P1, and P2 positions, P3 and P4 are farther from the shoulder, and the temperature increases at P3 and P4 are smaller, making the second stage indistinguishable from the first. As the tool pin moves further, the temperatures of these points decrease. At the peak temperature, the temperature field in the cross-section of the FSPed specimen presents the gradient in temperature (Figure 9c). The peak temperatures of P0 and P1 reach 1003.3 °C and 929.3 °C, respectively, which are higher than A_3_, indicating full austenitization of SZ. Therefore, after cooling to room temperature, martensite and a small amount of austenite form in SZ (Figure 3b). The peak temperature of P2 reaches 632.3 °C, within the range of A_1_–A_3_, leading to the formation of austenite and ferrite in TZ (Figure 3c). Additionally, a small amount of austenite remains in TZ due to the enrichment of C and Mn in the austenite (Figure 4b) [32]. P3 and P4 have peak temperatures below A_1_, leading to a tempering-like process in BM. During this process, a certain amount of austenite decomposes, leading to the formation of ferrite, fine austenite, and carbide (Figure 3e). Therefore, across the thickness direction from BM to TZ, and finally to SZ, a slight decreasing trend in austenite fraction is observed, forming a gradient in austenite fraction.

**Figure 10:**
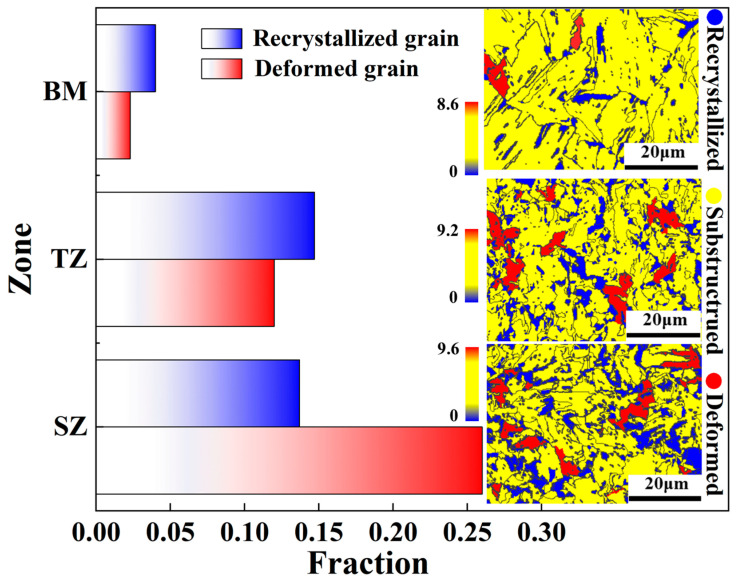
Volume fractions of deformed and recrystallized grains and grain orientation spread (GOS) diagram of BM, TZ, and SZ for the FSPed sample.

In addition to the austenite fraction gradient, a grain size gradient and dislocation density gradient across the thickness direction occur. Although no direct studies on the grain size gradient in FSPed medium Mn steel have been conducted, grain size refinement after friction stir welding has been observed [33,34]. These studies show that an increase in dynamically recrystallized grains can increase the degree of grain refinement during the hot-deformation process. In the present study, the volume fractions of recrystallized grains and the grain orientation spread (GOS) maps of BM, TZ, and SZ for the FSPed sample are shown in Figure 10. The GOS diagram can display the average misorientation between all data points in the grain, and grains with low misorientation angles indicate they have undergone recrystallization, while grains with high misorientation angles imply deformed grains [35,36]. According to the GOS maps, the volume fractions of recrystallized grains in TZ and SZ are much higher than in BM, which correlates well with the grain size gradient. However, compared to TZ, the volume fraction of recrystallized grains in SZ slightly decreases from 14.8% to 13.7%, while the grain size decreases significantly. This suggests that the dynamic recrystallized grains are not the only factor affecting grain size. According to Sun et al. [37], the severe plastic deformation during the FSP process, which results in the deformed sub-grains, also plays a role in grain refinement. In this study, the volume fraction of deformed grains in TZ is much lower than in SZ. Therefore, a small grain size is observed in SZ. In addition to the grain refinement, severe plastic deformation results in an increase in the dislocation density. The increasing sequence in the volume fraction of deformed grains from BM to TZ and finally to SZ represents the degree of severe plastic deformation, which corresponds to the increasing trend in dislocation density from BM to TZ and finally to SZ (Figure 5). All the gradients in grain size, austenite fraction, and dislocation density formed during the manufacturing process contribute to the hardness profile across the thickness direction (Figure 2).

### 4.2. Strengthening Mechanism for the Fabricated Medium Mn Steel

The main strengthening factors in homogeneous single-phase metallic materials include solid solution strengthening, fine grain strengthening, dislocation strengthening, and precipitation strengthening. Additional hetero-deformation-induced strengthening (HDI) strengthening is significant in materials with gradient, dual-phase, or multi-phase structures [38]. In the fabricated MMnS, no microalloying elements (such as Nb, V, Ti, etc.) are added and thus precipitation strengthening can be ignored. The yield strength and tensile strength of BM or SZ, excluding HDI strengthening, can be calculated by considering the contributions of each constituent phase. The volume fractions of austenite and ferrite in BM are 8.7% and 91.3%, respectively, while in SZ, the volume fractions of austenite and martensite are 2.2% and 97.8%, respectively. The calculation is based on mixing rules and is calculated as follows [39]:

(5) $$\sigma_{total}=\sum_{i}\sigma_{f}^{i}f_{f}^{i}$$

where $\sigma_{total}$, $\sigma_{f}^{i}$ and $f_{f}^{i}$ represent the total flow stress, the flow stress of the *i*-th phase (ferrite, martensite, and austenite), and the volume fraction of the *i*-th phase. The strength contribution of the investigated steel can be calculated by the following formula [3]:

(6) $$\sigma_{f}^{i}=\sigma_{0}+\sigma_{ss}+\sigma_{GB}+\sigma_{DIS}$$

where $\sigma_{0}$ is the lattice friction stress of pure *α*-Fe, $\sigma_{ss}$ is solid solution strengthening, $\sigma_{GB}$ is fine grain strengthening, and $\sigma_{DIS}$ is dislocation strengthening.

For solid solution strengthening, the $\sigma_{ss}$ of ferrite and martensite can be calculated by the following formula [40]:

(7) $$\sigma_{\alpha }^{ss}=1103.45x_{\alpha }^{C}+16.9x_{\alpha }^{Mn}$$

where $\sigma_{\alpha }^{ss}$ is the solid solution strengthening of martensite or ferrite, and $x_{\alpha }^{C}$ and $x_{\alpha }^{Mn}$ are the mass fractions of C and Mn in ferrite or martensite.

The $\sigma_{ss}$ of austenite can be calculated by the following formula:

(8) $$\sigma_{\gamma }^{ss}=598x_{\gamma }^{C}-1.4x_{\gamma }^{Mn}$$

where $\sigma_{\gamma }^{ss}$ is the solid solution strengthening of austenite. $x_{\gamma }^{C}$ and $x_{\gamma }^{Mn}$ are the mass fractions of C and Mn in austenite, respectively. In the FSPed specimen, the microstructures in SZ consist mainly of fresh martensite, a very small amount of retained austenite, and some martensite (Figure 2b and Figure 3b). The BM is composed of austenite and ferrite. The Mn concentrations in austenite and ferrite in the BM, as measured by TEM-EDS, are 3.48% and 4.98%, respectively. The Mn concentration in martensite in the SZ is measured to be 4.37%. The C concentrations in austenite and ferrite in the BM, calculated using formula (1) and the element conservation rule, are 0.90% and 0.045%, respectively. In the SZ, where the microstructure is mainly martensite with a small amount of retained austenite, it can be assumed that all the C concentration is distributed in the martensite [41]. Therefore, in the SZ, the C concentration in martensite is 0.12%.

The strength contribution of fine grain strengthening can be calculated by the following formula [42]:

(9) $$\sigma_{i}^{GB}=\frac{K_{i}}{\sqrt{d_{i}}}$$

where $\sigma_{i}^{GB}$ is the fine grain strengthening of the *i* phase. $K_{i}$ is a constant and equals 180 MPa·μm^1/2^ for austenite and 160 MPa·μm^1/2^ for ferrite or martensite [34]. $d_{i}$ is the effective grain size of the *i* phase.

The strength contribution of dislocation strengthening can be calculated as follows:

(10) $$\sigma_{i}^{DIS}=\alpha MGb\sqrt{\rho_{i}}$$

where $\sigma_{i}^{DIS}$ is the dislocation strengthening of the *i* phase, α is the geometric constant with a value of 0.25 [43], and M is the Taylor factor with a value of 2.73 [44]. G is the shear modulus, which is 76 GPa for ferrite or martensite and 65 GPa for austenite [45]. b is the Burger’s vector, with a value of 0.252 nm. $\rho_{i}$ is the average dislocation density of the *i*-th phase. The average dislocation densities of BM and SZ calculated by the modified W-H method when the true strain is 0.28 are 10 × 10^14^ m^−2^ and 13 × 10^14^ m^−2^, respectively.

**Figure 11:**
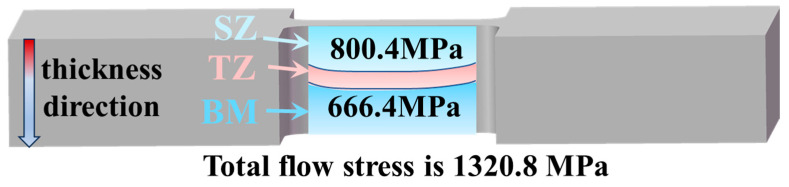
Comparison of flow stress contribution and total flow stress in different regions when the true strain is 0.28.

The determined strength contributions in SZ and BM, without accounting for HDI strengthening, are shown in Table 1. The strength contributions of both regions show that dislocation strengthening provides a significant contribution to flow stress. Moreover, the total flow stresses of BM and SZ, without accounting for HDI stress, are 666.4 MPa and 800.4 MPa, respectively. The hardness values in TZ are higher than those in BM but lower than those in SZ; hence, the total flow stress of TZ, without accounting for HDI stress, is higher than BM but lower than SZ. However, the strength contribution of the individual BM, TZ, or SZ regions cannot meet the measured overall flow stress (Figure 11). Even if SZ contributes to the total flow stress, the lowest HDI flow stress of 520.4 MPa is needed. Therefore, the contribution to the flow stress from total HDI strengthening, including gradient structure HDI strengthening and multiphase HDI strengthening, is likely the main factor affecting the mechanical properties.

**Figure 12:**
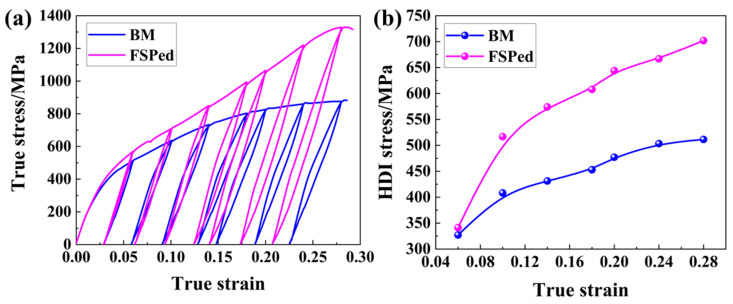
LUR test curves and the determined HDI stress in the base material and FSPed specimen. (**a**) LUR true stress–strain curves; (**b**) HDI stress under different strains.

In this study, the HDI stress can be evaluated using the loading–unloading–reloading (LUR) test, and total HDI stress can be calculated by the following formula [46]:

(11) $$\sigma_{HDI}=\frac{\sigma_{u}+\sigma_{r}}{2}$$

where $\sigma_{u}$ and $\sigma_{r}$ are the unloading yield and load yield stress, respectively. The LUR test curves and the corresponding HDI stress in the base material and FSPed specimen are revealed in Figure 12. The base material serves as a benchmark because its HDI strengthening results solely from the multi-phase structure. As shown in Figure 12, the HDI stress of the FSPed sample is significantly higher than that of the base material, directly indicating the HDI strengthening due to the gradient structures. Moreover, at a true strain of 0.28, the HDI stress is approximately 701.9 MPa, significantly higher than the 520.4 MPa (the lowest HDI flow stress needed), indicating that HDI strengthening cannot be solely attributed to the SZ. As revealed in Figure 9, a significant increase in hardness values is observed in TZ during the deformation process. Moreover, TZ has a much higher increment in hardness and the highest dislocation density, indicating that significant HDI stress forms in TZ. Therefore, TZ is the primary zone where HDI stress forms. It should be noted that the formed HDI stress not only results from the strength and hardness difference of different zones but also the inherent difference in properties among the multiple phase domains in the same zone.

**Figure 13:**
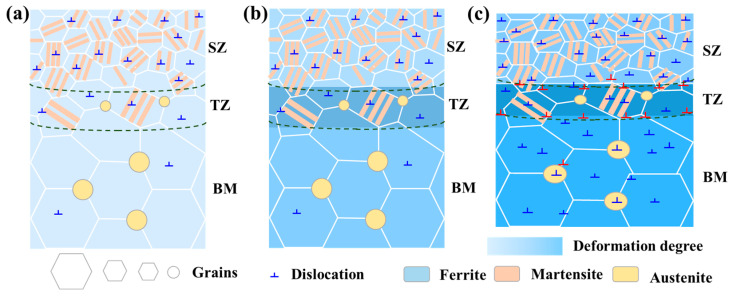
Schematic diagram showing the deformation mechanism of the investigated steel during the deformation process: (**a**) Before tensile deformation; (**b**) Early stage of tensile deformation; (**c**) Late stage of tensile deformation.

A schematic diagram illustrating the deformation mechanism of the fabricated steel using FSP technology is finally summarized and shown in Figure 13. Prior to the deformation, gradient structures, including the grain size gradient, austenite fraction gradient, and dislocation density gradient across the thickness direction, form (Figure 13a). During the tensile-deformation process, the microstructure of each region deforms, but more severe deformation occurs in TZ (Figure 13b). Heterogeneous deformation occurs across the thickness direction, with additional dislocations forming in the TZ, resulting in hetero-deformation-induced strengthening (Figure 13c). The total hetero-deformation-induced strengthening and the dislocation strengthening are the main factors for the FSPed medium Mn steel achieving excellent strength–ductility synergy.

## 5. Conclusions

In this work, FSP manufacturing technology was used to fabricate medium manganese steel with excellent mechanical properties. Microstructure for the formation of gradient structure during the manufacturing process was characterized. The changes in the mechanical performance before and after FSP were analyzed. The main conclusions are as follows:

(1). FSP manufacturing technology can increase the tensile strength of medium Mn steel from 650.7 MPa to 1034.6 MPa while slightly reducing the total elongation from 38.3% to 35.1%. The product of tensile strength and elongation of the medium Mn steel increases significantly from 24.9 GPa∙% to 36.3 GPa∙%.
(2). Decreasing trends of grain size and austenite fraction, as well as an increasing trend of the dislocation density, are obtained across the thickness direction from the base material zone to the transition zone and finally to the stirring zone, which results from the simultaneous effect of severe plastic deformation and recrystallization during friction stir processing.
(3). Hetero-deformation-induced strengthening and dislocation strengthening are the main factors for the elevated mechanical properties of the fabricated steel. The transition zone has the obvious hetero-deformation-induced stress during the deformation process, and the determined flow stress contributed from the hetero-deformation-induced stress is about 701.9 MPa with a true strain of 0.28.

## Figures and Tables

**Table 1:** Stress contribution of different strengthening mechanisms of BM and SZ when the true strain is 0.28.

Zone	BM	SZ
σ_0_ (MPa)	78.1	78.1
σ_SS_ (MPa)	145.2	205.1
σ_GB_ (MPa)	56.7	82.4
σ_DIS_ (MPa)	386.4	434.9

## Data Availability

Data are contained within the article.

## Supplementary Materials

The following supporting information can be downloaded at: https://www.mdpi.com/article/10.3390/mi15081052/s1, Figure S1: Grain size distribution maps in different zones; Figure S2: Band contrast maps and phase maps of the BM and SZ zones.

## Nomenclature

FSP	Friction stir processing.
HDI	Hetero-deformation-induced.
MMnS	Medium manganese steels.
TWIP	Twinning-induced plasticity.
A1	Austenite starting temperature.
A3	Austenite finish temperature.
SZ	Stirring zone.
TZ	Transition zone.
BM	Base material zone.
FEA	Finite element analysis.
CLSM	Confocal laser scanning microscope.
FE-SEM	Field-emission scanning electron microscope.
EBSD	Electron backscatter diffraction.
TEM	Transmission electron microscope.
EDS	Energy-dispersive spectroscopy.
XRD	X-ray diffraction.
IPF	Inverse pole figure.
BC	Band contrast.
KAM	Kernel average misorientation.
GND	Geometrically necessary dislocation.
YS	Yield strength.
UTS	Ultimate tensile strength.
TE	Total elongation.
SMAT	Surface mechanical attrition treatment.
USSR	Ultrasonic severe surface rolling.
LUR	Loading–unloading–reloading.
