# Supplementary material for: Fabrication of Medium Mn Advanced High-Strength Steel with Excellent Mechanical Properties by Friction Stir Processing
Source: Micromachines (Basel). 2024 Aug 21;15(8):1052. doi: 10.3390/mi15081052 (PMC11356244; doi:10.3390/mi15081052)
Supplement: Supplementary file 1 [file micromachines-15-01052-s001.zip › micromachines-3073912-supplementary.pdf]

Fabrication of Medium Mn Advanced High-Strength Steel with Excellent Mechanical Properties by Friction Stir Processing

Yonggang Yang [1], Wangnan Zuo [1], Yu Liu [2,*], Yunzong Ge [3], Zhiqiang Yang [3], Jiansheng Han [4] and Zhenli Mi [1,*]

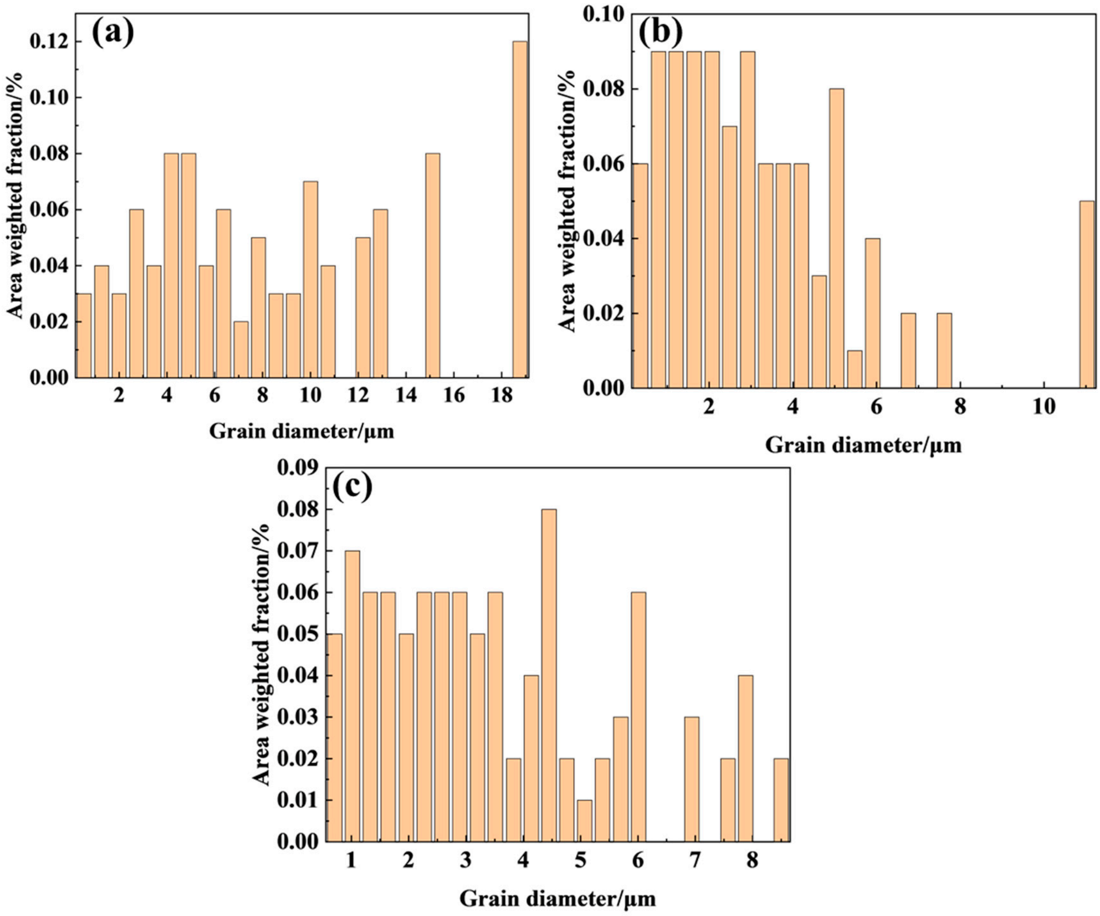

Figure S1 Grain size distribution maps in different zones. (a) BM (b) TZ, and (c) SZ.

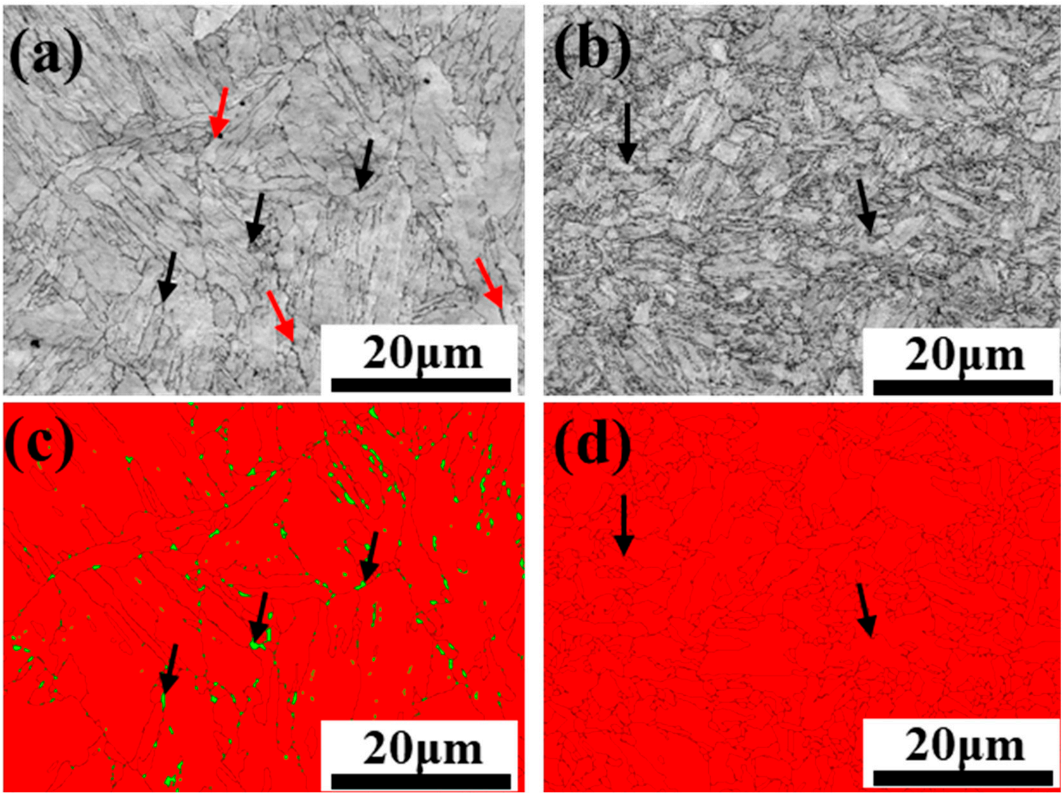

Figure S2 Band contrast maps and phase maps of the BM and SZ zones. (a) Band contrast map of the BM (Zero resolution: 5.3%), (b) Band contrast map of the SZ (Zero resolution: 7.8%), (c) Phase map of the BM, and (d) Phase map of the SZ.
